# General and ICT Self-Efficacy in Different Participants Roles in Cyberbullying/Victimization Among Pakistani University Students

**DOI:** 10.3389/fpsyg.2019.01098

**Published:** 2019-05-14

**Authors:** Sadia Musharraf, Sheri Bauman, Muhammad Anis-ul-Haque, Jamil Ahmad Malik

**Affiliations:** ^1^National Institute of Psychology, Quaid-i-Azam University, Islamabad, Pakistan; ^2^Department of Applied Psychology, The Women University Multan, Multan, Pakistan; ^3^Disability and Psychoeducational Studies, The University of Arizona, Tucson, AZ, United States; ^4^Department of Applied Psychology, National University of Modern Languages (NUML), Islamabad, Pakistan

**Keywords:** traditional bullying, traditional victimization, cyberbullying, cyber victimization, ICT self-efficacy, general self-efficacy, Pakistan, university students

## Abstract

The study examines both general and Internet and Communication Technology (ICT) self-efficacy in cyber-victims, cyber-bullies, and cyber bully victims in comparison to un-involved students. Gender differences were also examined. A total of 1115 Pakistani university students from six universities participated in the study. Analyses were conducted on 950 complete cases (371 males, and 579 females). Data were collected on cyberbullying/victimization, general self-efficacy (GSE), ICT self-efficacy, traditional bullying/victimization, ICT usage, social desirability, and demographics. Multinomial logistic regression analysis indicated that ICT self-efficacy significantly decreased the probability of being a cyber-victim and significantly increased the chances of being a cyber-bully whereas GSE appeared to have no role in predicting participant roles in cyberbullying after controlling for covariates (i.e., age, gender, traditional bullying, traditional victimization, social desirability, Internet usage, time spent on the Internet, and social networking sites (SNS). Findings of the study have important implications for developing and enhancing interventions with respect to the inclusion of ICT related skills in anti-cyberbullying programs. With respect to gender, findings showed that females reported a higher level of victimization while males reported higher perpetration on both traditional and cyberbullying.

## Introduction

Rapid development of Internet Communication Technology (ICT) has facilitated opportunities for developing social connections, fast digital interactions, and the availability of interactive and self-directed learning (Mishna et al., 2018). Access to information and dissemination of information to a larger audience have become easy with the use of Internet and social media tools (Heirman and Walrave, 2008; Patchin and Hinduja, 2011). Although ICT and fast-growing digital technologies have provided many benefits to students in higher education, these technologies also have a darker side that can be used to inflict harm to others (Mishna et al., 2010; Musharraf and Lewis, 2016). One such harmful behavior is cyberbullying.

Cyberbullying is “any behavior performed through electronic or digital media by individuals or groups that repeatedly communicates hostile or aggressive messages intended to inflict harm or discomfort on others” (Tokunaga, 2010) that involves a power imbalance between a target and the perpetrator (Olweus, 1993; Kowalski et al., 2014). Cyberbullying can be inflicted through text messages, websites, emails, blogs, chat rooms, social networking sites (SNS), digital apps such as Instagram, Twitter, Facebook, Skype, WhatsApp, Snapchat, video sharing platforms, and game servers, etc. (Cassidy et al., 2018). These behaviors include sending intimidating, threatening shameful and harassing messages, posting false rumors about the target, sharing private and sensitive information about others online, stealing someone’s online identity by creating fake profiles and web pages, and deliberately excluding someone from an online group (Li, 2007; Willard, 2007; Nocentini et al., 2010).

Cyberbullying emerged over the past two decades with the globalization of the internet (Festl, 2016), and has become an international public health issue not only for children and adolescents but also for adults (Nixon, 2014). Research has found that cyberbullying victimization among university students is associated with wide-ranging devastating psychological and mental health problems including, anxiety, stress, depression, irritability, helplessness, loneliness, sleep disturbances, and difficulties in maintaining concentration (Faucher et al., 2014; Cassidy et al., 2017; Musharraf and Lewis, 2018) and in more extreme situations even suicidal ideation (Schenk and Fremouw, 2012; Mitchell et al., 2018). Furthermore, cyberbullying perpetration in this population is associated with low empathy, high impulsivity, hostility, depression, and psychoticism (Arıcak, 2009; Doane et al., 2014; Kokkinos et al., 2014). Despite the serious impacts of cyberbullying/victimization among university students, less attention has been given to investigate this phenomenon among university students and only few studies have investigated its association with individual level variables such as self-efficacy. Further, inconsistent findings have been found concerning gender differences in cyberbullying (Larrañaga et al., 2016).

There is a controversy in the literature over whether cyberbullying is an extension of the traditional form of bullying or a unique and separate phenomenon (Antoniadou and Kokkinos, 2015). There are two important considerations in favor of the former position. First, both traditional and cyberbullying often occur together (Beran and Li, 2005), and a high degree of overlap exists in the involvement of students in traditional and cyberbullying (Olweus, 2012a, b). Second, both types of bullying share common risks and protective factors (Hase et al., 2015; Völlink et al., 2016).

One such protective factor is self-efficacy (Sapouna and Wolke, 2013). Self-efficacy is a component of Albert Bandura’s Social Cognitive Theory (e.g., Bandura, 1994, 2008). Self-efficacy refers to an individuals’ judgment of his or her personal capabilities to achieve designated goals, organize and perform a course of action, and regulate one’s psychological functioning (Bandura, 1997). Prior research concerning traditional bullying victimization has revealed a negative association between victimization and general self-efficacy (GSE) among school students (Erath et al., 2010; Kokkinos and Kipritsi, 2012). However, inconsistent findings have been reported in the literature with reference to GSE and traditional bullying perpetration. For example, Natvig et al. (2001) found a significant positive association between self-efficacy and perpetration of bullying among adolescents, and this association was stronger for older students. In contrast, others found a negative association between GSE and the perpetration of bullying (Kokkinos and Kipritsi, 2012).

This line of inquiry has been further extended to examine the relationship of self-efficacy to cyberbullying victimization and perpetration. For instance, a study by Olenik-Shemesh and Heiman (2014) showed that a lower level of social and emotional self-efficacy was found in cyber victims than in non-victims. Other studies found a negative association between GSE and both cyberbullying victimization and perpetration (Wong et al., 2014; Eden et al., 2016). The construct of self-efficacy is highly domain-specific instead of a general disposition, and thus changes across circumstances, settings, and situations (Bandura, 1986). In view of this, Bussey et al. (2015) found that self-efficacy beliefs to engage in cyberbullying were positively associated with cyberbullying perpetration.

Considering a domain-specific approach, it is imperative to investigate the role of ICT self-efficacy in cyberbullying and victimization. Until recently, a few studies focused on ICT self-efficacy with reference to cyberbullying, and inconsistent findings have been reported. For example, Xiao and Wong (2013) found that Internet self-efficacy has a significant positive impact on the perpetration of cyberbullying. Similarly, a significant positive association between Internet self-efficacy and the perpetration of cyberbullying was detected in a study by Musharraf et al. (2018). Conversely, Savage and Tokunaga (2017) found that Internet self-efficacy was not associated with cyberbullying perpetration; however, verbal aggression moderated the relationship between social skills and cyberbullying perpetration only for those who possess high Internet self-efficacy. This study did not examine the relationship of cyber victimization with Internet self-efficacy. Additionally, Internet self-efficacy was measured by a scale (Eastin and LaRose, 2000) that only contains items relating to Internet software, hardware, and trouble shooting. Hence, these items may not reflect the skills used in cyberbullying.

Besides these inconsistent findings, there is a clear lack of research with reference to ICT self-efficacy and cyber victimization. Further, to date no study examined the comparative role of General and ICT self-efficacy in predicting cyberbullying and victimization. Bullying has been considered a group process and students involved in bullying or cyberbullying may assume different roles such as bully, victim, bully victim and un-involved (Salmivalli et al., 1996). The current research therefore was conducted to bridge the gap by examining the comparative role of general and ICT self-efficacy in determining the involvement of university students in different cyberbullying roles.

Existing research indicated several potential factors that may attribute to the variation in reports of cyberbullying/victimization (Betts, 2016). For example, variations in the prevalence rates have been reported with reference to the characteristics of the sample of the study such as their gender, age, usage of ICT, and amount of time spent online. Inconsistent findings have been reported concerning gender and cyberbullying/victimization. Some studies demonstrated greater victimization of males than females (Wensley and Campbell, 2012) while others reported greater victimization of females in comparison to males (Paullet and Pinchot, 2014; Caravaca-Sanchez et al., 2016; Webber and Ovedovitz, 2018). A number of studies found no gender differences (MacDonald and Roberts-Pittman, 2010; Wozencroft et al., 2015; Gibb and Devereux, 2016). Further, several studies found males outnumbered females for cyberbullying perpetration (Ballard and Welch, 2017), who were involved more as victims and bullies (Akbulut and Eristi, 2011; Wong et al., 2018) and as bullies and mixed victim-bullies (Cunningham et al., 2015; Kokkinos et al., 2016) in comparison to females. Conversely, some studies indicated females were found to be higher in performing cyberbullying behaviors (Schenk et al., 2013) and more involved as both victims and bullies in comparison to males (Francisco et al., 2015).

With reference to age, it has been found that younger students experience more cyberbullying than the older students (Ševčíková and Šmahel, 2009; Zalaquett and Chatters, 2014). Further, research demonstrated that higher ICT usage, more time spent online generally, or particularly on social media may influence the likelihood with which one experiences cyberbullying or performs cyberbullying perpetration (Livingstone and Helsper, 2010; Leung and Lee, 2012; Zhou et al., 2013; Navarro et al., 2017).

Olweus (2012a, b, 2013) recommended researchers to measure cyberbullying contemporarily in the broader context of traditional bullying. This allows researchers to contextualize the level of normative aggression within a particular sample. Further, he cautioned researchers that associated harms of cyberbullying/victimization should not be taken without considering the co-existing harms of traditional bullying/victimization (Olweus, 2012a, b). In addition, social desirability is another potential factor that can lead to over-reporting or under-reporting or of cyberbullying/victimization. University students generally consider cyberbullying as socially undesirable behavior (Akbulut and Eristi, 2011; Betts, 2016) and existing research reported a positive association between cyberbullying and social desirability (Doane et al., 2013).

In view of all this, the well-established covariates such as age, gender, concurrent involvement in traditional bullying and victimization, ICT usage, time spent on the Internet, time spent on SNS and social desirability (Betts, 2016; Wolke et al., 2016), were controlled for the precise estimation of the comparative role of general and ICT self-efficacy in determining the involvement of students in different roles of cyberbullying among Pakistani university students.

## Materials and Methods

### Sample

The sample for this study was comprised of 1115 Pakistani university students from six different universities. Only complete cases were included in the analyses using listwise deletion for handling missing which resulted in 950 valid cases. Listwise deletion means that if a participant had missing data on any variable in the analysis, their case was removed. Age of the participants ranged from 18 to 25 years with Mean ± 20.79 and *SD* = ±1.94. Out of the total sample, 39.05% were males and 60.95 % were females. A total of 57% participants were enrolled in undergraduate, and 43% in masters programs. Further, 73.97% participants were enrolled in social sciences and arts, and the remaining 24.96% were from natural sciences disciplines.

### Measures

#### Cyberbullying and Cyber Victimization Scales

Following existing research (Del Rey et al., 2015), and findings of a qualitative study (Musharraf et al., 2018), the scales were developed by the first author and validated on a sample of Pakistani university students (Musharraf and Anis-ul-Haque, 2018b) Each of the cyberbullying and cyber victimization scales included 20 Likert-type items with response options ranging from (0) “Never” to (4) “More times a week.” Sample items include: “Someone posted my private pictures or videos online in a mean or hurtful way” for cyber victimization scale and “I posted someone’s private pictures or videos online in a mean or hurtful way” for cyber bullying scale.

The time frame of “past 12 months” was used to ask respondents about the frequency of cyberbullying and cyber victimization. The scales are scored in two different ways. A sum of the scores on all items of each scale represents an overall score on that scale and high scores on each scale signifies higher levels of cyberbullying and cyber victimization, respectively (Musharraf and Anis-ul-Haque, 2018b). The overall scores on each scale can be used as a continuous score. Further, the scores on the scale can be used to categorize individuals into one of the four groups: cyber victims, cyber bullies, cyber bully victims, and un-involved (see Musharraf and Anis-ul-Haque, 2018a). Following Del Rey et al. (2015), this categorization was made on the basis of behaviors participation and repetition in a particular role. Thus, cyber-victims were those participants who scored equal or higher than (2) “once a month” in any of the items of cyber victimization scale and with scores equal or lower than (1) “once or twice” in all of the items of cyber bullying scale. Cyber bullies were identified as those subjects who scored equal or higher than (2) “once a month” in any of the items of cyber bullying scale and equal or lower than (1) “once or twice” in all of the items of cyber victimization scale. Cyber bully/victims were those participants who scored equal or higher than (2) “once a month” in any of the items of both cyber victimization and cyber bullying.

For the present study, both continuous and categorical scores were used. The continuous scores were used to conduct preliminary analysis, whereas the categorical scores were used for the classification into groups by the different roles in cyberbullying. The scale has good internal consistency; Cronbach’s alphas 0.83 to 0.85 were reported (Musharraf and Anis-ul-Haque, 2018b), and for the present study Cronbach’s alphas 0.83 and 0.86 were found for the Cyberbullying and Cyber Victimization Scales, respectively.

#### General Self-Efficacy (GSE)

General Self-efficacy was assessed by ten-item scale (Schwarzer and Jerusalem, 1995). The scale has response options on a 4-point Likert scale ranging from 1 (Not at all true) to 4 (Exactly true). The overall score on the scale was determined by summing all the items and scores ranged from 10 to 40; higher scores signify higher levels of GSE. Example items include: “I am confident that I could deal efficiently with unexpected events” and “If I am in trouble, I can think of a good solution.” The scale has good reliability with alpha coefficients ranging from 0.76 to 0.90 (Schwarzer and Jerusalem, 1995). Alpha coefficient of 0.88 showed high internal consistency of the scale for the present study sample. The validity of the scale was established by finding positive correlations of the total score on the GSE to work satisfaction, and optimism and the negative correlations to stress, depression, burnout, and anxiety (Schwarzer and Jerusalem, 1995).

#### ICT Self-Efficacy Scale

ICT Self-efficacy Scale is a self-report measure comprised of 18 items and scored on a 5-point Likert type scale ranging from (1) “Disagree strongly” to (5) “Agree strongly.” The overall score on the scale is obtained by summing all the items. A higher score on the scale indicates a higher level of ICT-self-efficacy. Sample items include: “I can easily recover my email /social networking account if I forget the password” and “I can easily report a fake account pretending to be me.”

The scale was developed and validated on university students and has good internal consistency with alphas ranging from 0.92 to 0.93 (Musharraf et al., 2018) and alpha 0.92 was found for the present study.

#### California Bully Victimization Scale (CBVS)

Traditional bullying and victimization were measured by CBVS (Felix et al., 2011), that originally consisted of 16 items that measure traditional bullying and victimization in students. Eight items measure traditional victimization by asking the respondents to rate their responses on a five-point Likert scale ranging from (0) “Never” to (4) “More times a week.” Similarly, a parallel set of eight items measures traditional bullying. For the present study, two items related to the measurement of cyberbullying and cyber victimization were removed because we used a separate scale for this measurement. The time frame of “past 12 months” was used to measure the frequency of bullying and victimization. Example items include: “How often have you been threatened in a mean or hurtful way?” for victimization dimension and “How often have you threatened another student in a mean or hurtful way?” for bullying dimension. Atik and Guneri (2012) reported satisfactory internal consistency ranged from 0.72 to 0.83 for the scale. For the present study sample, the scales showed good internal consistency with alpha 0.79, and 0.83 for victimization dimension and for bullying dimension, respectively.

#### ICT Use Scale

The ICT Use scale was originally developed to measures adolescents’ ICT use (Sticca et al., 2013). The scale was adapted to use in the present study with reference to university-aged students and consists of 16 statements tapping the frequency of online activities. Examples of these activities include: phone calls, chatting, posting information online, playing computer, or video games, etc. Respondents were asked to report how often they had performed these activities in the past 12 months. Responses options are on a five-point Likert scale ranging from (1) “never” to (5) “almost daily.” The scores are summed to find an overall score, and a higher score indicates higher ICT use. The scale showed good internal consistency with alpha reliability 0.93 for the present sample. Along with online activities, respondents were also asked to report the average duration of time spent online on any weekday, on weekends (i.e., Sunday or holiday), and time spent on SNS.

#### Social Desirability Scale (SDS)

The social desirability scale (SDS) is a 16-item scale measuring behaviors that are considered socially desirable. The scale has a dichotomous response style with (0) “False” and (1) “True.” Six items are reversed scored. Items are summed to get an overall score on the scale and high score on the scale represent a higher level of social desirability in respondents. Sample items include: “I occasionally speak badly of others behind their back” and “During arguments I always stay objective and matter-of-fact.” The scale is a valid and reliable measure of the social desirability and Cronbach’s alpha reliability ranged from 0.72 to 0.80 in various studies (Stöber, 2001). Cronbach’s alpha reliability of 0.77 was found for the present study.

### Procedure

Before data collection, the proposal for the study was evaluated and approved by the Ethical Review Board of the National Institute of Psychology, Quaid-i-Azam University, Islamabad, against the American Psychological Association ethical guidelines. Participants were approached at their respective universities. Participants were briefed about the objectives of the study and both verbal and written consent were taken. Students who agreed to participate were then asked to sign a voluntary consent form before their participation in the study. An anonymous survey composed of the measures described above was administered to participants in a group setting during class hours.

## Results

The present study was designed to investigate the comparative role of general and ICT self-efficacy in determining the different roles in cyberbullying. Following existing research by Del Rey et al. (2015), respondents ratings on cyber bullying and victimization scales were used to categorize participants into cyber-bullies (*n* = 66), cyber-victims (*n* = 286), cyber bullies-victim (*n* = 260), and un-involved (*n* = 338). Sum scores on cyberbullying and victimization scale were used for preliminary analysis. Results presented in Table 1, showed that age was negatively correlated with both traditional victimization (*r* = −0.06, *p* < 0.05), and cyber victimization (*r* = −0.09, *p* < 0.01) suggesting that younger students are more likely to be victimized. Gender appeared to be positively correlated with traditional and cyber victimization (*r* = 0.20, and 0.29, respectively, *p* < 0.01) and negatively correlated with both traditional and cyberbullying (*r* = 0.24, and 0.25, respectively, *p* < 0.01). These results suggested that females are more vulnerable to be victims both in traditional and cyber contexts whereas males are more prone to bullying perpetration. Time variables (i.e., average time spent on the internet on a weekday, weekend, and average time spent on SNS) as well as ICT usage were positively correlated with both cyber victimization (*r* range = 0.16 to 0.29, *p* < 0.01), and cyberbullying (*r* range = 0.16 to 0.59, *p* < 0.01). These results suggest that spending more time on the Internet and particularly on SNS is associated with increased cyberbullying perpetration as well as increased risk of cyber victimization. Finally, cyberbullying perpetration was positively correlated with ICT self-efficacy (*r* = 0.06, *p* < 0.05) whereas it was negatively correlated with GSE (*r* = −0.12, *p* < 0.01) indicating that ICT self-efficacy may increase cyberbullying perpetration whereas GSE may be a protective factor that decreases the likelihood of indulging in cyberbullying perpetration.

**TABLE 1:** Pearson bivariate correlations among study variables (*N* = 950).

		k	Alpha	1	2	3	4	5	6	7	8	9	10	11	12	13
1	Age	–	–	−	$-0.227^{**}$	-0.025	0.029	-0.020	$-0.061^{*}$	0.010	$-0.091^{**}$	0.003	$-0.086^{**}$	0.034	0.026	-0.030
2	Gender	–	–		−	-0.005	-0.007	-0.008	$0.201^{**}$	$-0.235^{**}$	0.049	$-0.100^{**}$	$0.287^{**}$	$-0.245^{**}$	-0.006	$0.062^{*}$
3	TS-SNS	–	–			−	$0.361^{**}$	$0.502^{**}$	$0.181^{**}$	$0.179^{**}$	0.015	$0.235^{**}$	$0.280^{**}$	$0.202^{**}$	0.026	$-0.073^{*}$
4	TS-Weekdays	–	–				−	$0.466^{**}$	$0.187^{**}$	$0.087^{**}$	$-0.069^{*}$	$0.152^{**}$	$0.159^{**}$	$0.159^{**}$	$0.098^{**}$	$-0.073^{*}$
5	TS-Weekends	–	–					−	$0.205^{**}$	$0.107^{**}$	-0.013	$0.333^{**}$	$0.265^{**}$	$0.239^{**}$	$0.138^{**}$	-0.003
6	Traditional Victimization	7	0.79						−	$0.334^{**}$	-0.015	$0.129^{**}$	$0.517^{**}$	$0.277^{**}$	0.004	$-0.064^{*}$
7	Traditional Bullying	7	0.83							−	-0.008	$0.092^{**}$	$0.280^{**}$	$0.588^{**}$	$-0.081^{**}$	$-0.130^{**}$
8	Social Desirability	16	0.77								−	-0.013	-0.005	$-0.094^{**}$	-0.042	$0.118^{**}$
9	ICT Usage	16	0.93									−	$0.214^{**}$	$0.151^{**}$	$0.246^{**}$	$0.113^{**}$
10	Cyber victimization	20	0.83										−	$0.372{}^{**}$	0.017	-0.039
11	Cyber bullying	20	0.86											−	$0.056^{*}$	$-0.116^{**}$
12	ICT self-efficacy	18	0.92												−	$0.495^{**}$
13	General self-efficacy	10	0.88													−
	*M*			20.839	−	2.737	2.462	5.021	4.601	2.154	11.190	58.379	8.873	4.036	60.769	28.390
	*SD*			1.901	−	2.280	2.036	3.101	5.221	3.460	2.873	10.862	7.836	6.274	14.518	6.354

*p < 0.05, ^∗∗^*p* < 0.01. k, number of items; TS, Average time spend; SNS, Social Networking Sites; and ICT, Internet and Communication Technology.

Gender differences as presented in Table 2 further confirmed the correlational findings, suggesting that both traditional and cyber victimization are higher in females as compared to male students. Female participants reported on average 2.14 (*p* < 0.01) points higher rates of traditional victimization and 6.15 (*p* < 0.01) points higher cyber victimization compared to their male counterparts. In contrast, males scored higher than females on both traditional bullying (Mean difference = 1.65; *p* < 0.01) and cyberbullying (Mean difference = 3.13; *p* < 0.01). These results also indicated that the risk for female victimization is almost three times greater and bullying perpetration of males is two times greater in the cyber context. Considering that girls were significantly younger than boys and that younger students were more vulnerable to both traditional and cyber victimization, we further extended our analysis to estimate unique role of gender by controlling confounding effect of age. Partial correlations of gender with traditional bullying and victimization and cyberbullying and victimization were computed controlling for the effect of age. The results showed very little change in partial correlation coefficient from zero-order correlation coefficient for traditional victimization (*r*_0_ = 0.20 to *r*_p_ = 0.19) and cyber victimization (*r*_0_ = 0.29 to *r*_p_ = 0.28) and no change in traditional bullying and cyberbullying. These results further provided evidence of gender differences in the prevalence of bullying and victimization both in traditional and cyber context. Though no significant differences emerged on time variables (i.e., average time spend on the Internet on a weekday, weekend, and average time spend on SNS), ICT usage was significantly higher (Mean difference = 2.21, *p* < 0.01) in male university students in comparison to their female counterparts. Similarly, no significant gender differences appeared on ICT self-efficacy, yet females scored on average 0.80 (*p* < 0.05) points higher on GSE.

**TABLE 2:** Mean differences in study variables across gender (*N* = 950).

	Males		Females				95% *CI*	
	*M*	*SD*	*M*	*SD*	*t*	*p*	*LL*	*UL*
Age	21.360	2.018	20.483	1.730	8.425	0.000	0.673	1.081
TS-SNS	2.752	2.130	2.727	2.382	0.188	0.851	-0.239	0.290
TS-Weekdays	2.481	1.870	2.450	2.141	0.242	0.809	-0.217	0.278
TS-Weekends	5.050	2.928	5.001	3.214	0.276	0.783	-0.299	0.397
Traditional Victimization	3.335	3.024	5.470	6.153	-7.425	0.000	-2.699	-1.571
Traditional Bullying	3.133	4.212	1.481	2.628	8.739	0.000	1.281	2.022
Social Desirability	10.913	2.934	11.445	2.760	-1.774	0.076	-0.490	0.025
ICT Usage	59.685	11.340	57.480	10.433	3.631	0.000	1.014	3.397
Cyber Victimization	5.604	5.102	11.758	7.258	-10.860	0.000	-7.266	-5.043
Cyber Bullying	5.890	7.786	2.764	4.564	9.150	0.000	2.456	3.797
ICT Self-efficacy	60.869	15.862	60.703	13.563	0.199	0.842	-1.469	1.802
General Self-efficacy	27.918	6.682	28.716	6.100	-2.233	0.026	-1.500	-0.097

*TS, Average time spend; SNS, Social Networking Sites; and ICT, Internet and Communication Technology.*

**FIGURE 1:**
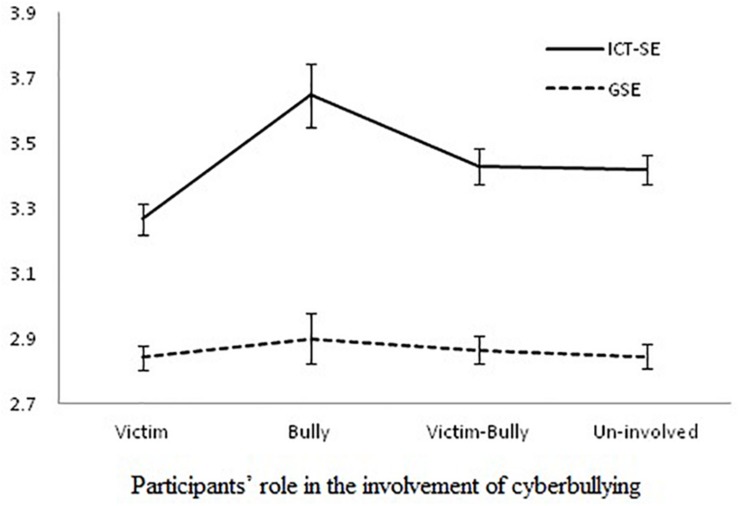
Comparison of general self-efficacy and ICT self-efficacy for participants’ role in the involvement of cyberbullying.

Multinomial logistic regression analysis was conducted to test the role of general and ICT self-efficacy in the different roles in cyberbullying (the categorical outcome variables based on Del Rey et al. (2015) classification) while controlling for the effect of covariates (i.e., age, gender, average time spent on the Internet on a weekday, weekend, average time spent on SNS, ICT usage, traditional bullying, traditional victimization, and social desirability). The probability of students being classified as cyber-victims, cyber-bullies, and cyber bullies-victim was predicted in reference to the un-involved group. The log-likelihood of the model significantly decreased (*χ*^2^ = 520.53, *p* < 0.01) from the baseline model suggesting that the regression model explained a significant amount of variance in different roles of cyberbullying. Both Pearson and Deviance statistics appeared to be non-significant, suggesting that model is a good fit to the data. The model explained substantial variance, with Cox and Snell *R*^2^ = 0.42 (Cox and Snell, 2018), and Nagelkerke’s *R*^2^ = 0.46 (Nagelkerke, 1991). The results presented in Table 3 show that ICT self-efficacy significantly decreased the probability of being a cyber-victim (*B* = −0.02, *p* < 0.01) and significantly increased the chances of being a cyber-bully (*B* = 0.03, *p* < 0.05). The odds ratios further showed that each unit decrease in ICT self-efficacy increased the chances of being a cyber-victim by 1.02 times and each unit increase in ICT self-efficacy increased chances of becoming a cyber-bully by 1.03 times. Neither general nor ICT self-efficacy significantly predicted being a cyber bully victim. A comparison of both general and ICT self-efficacy in determining the different roles in cyberbullying is further illustrated in Figure 1, which was developed using estimated marginal means and standard errors of both general and ICT self-efficacy. The marginal means and standard errors were estimated using MANCOVA for controlling the effect of covariates. The graph shows a non-significant role of GSE in all roles of cyberbullying. In contrast, a significant decrease in ICT self-efficacy is associated with being a cyber-victim and a significant increase in ICT self-efficacy is associated with being a cyber-bully.

**TABLE 3:** Logistic regression coefficient and odd ratios to predict different roles in cyberbullying (*N* = 950).

	Victim			Bully			Victim-Bully		
	B	*p*	Exp(B)	B	p	Exp(B)	B	*p*	Exp(B)
Intercept	-5.327	0.000		-1.805	0.413		-5.890	0.000	
Age	0.058	0.234	1.060	-0.043	0.584	0.958	0.049	0.402	1.050
Gender	0.889	0.000	2.432	-1.773	0.000	0.170	-0.127	0.603	0.881
TS-SNS	0.139	0.007	1.149	-0.163	0.080	0.850	0.107	0.057	1.113
TS-Weekdays	0.109	0.050	1.116	0.015	0.867	1.015	0.084	0.163	1.087
TS-Weekends	0.013	0.731	1.013	0.212	0.000	1.236	0.215	0.000	1.240
Traditional Victimization	0.101	0.000	1.106	0.133	0.001	1.142	0.174	0.000	1.190
Traditional Bullying	0.127	0.017	1.135	0.246	0.000	1.279	0.348	0.000	1.416
Social Desirability	-0.010	0.791	0.990	-0.058	0.360	0.944	-0.061	0.174	0.941
ICT Usage	0.035	0.000	1.036	0.019	0.232	1.019	0.033	0.004	1.034
ICT Self-efficacy	-0.022	0.004	0.978	0.030	0.029	1.031	-0.001	0.910	0.999
General Self-efficacy	0.025	0.130	1.026	-0.019	0.520	0.981	0.011	0.592	1.011

*TS, Average time spend; SNS, Social Networking Sites; and ICT, Internet and Communication Technology.*

## Discussion

Despite the growing evidence about the high prevalence and deleterious impacts of cyber bullying, less is known about the predictive factors particularly germane to cyberbullying. Most previous studies focused on extending the application of existing knowledge about traditional bullying to cyberbullying instead of examining the factors particularly relevant to cyberbullying with respect to digital context and online spaces. Therefore, the present aim was to examine the comparative role of general and domain-specific ICT self-efficacy in predicting the involvement of university students in various roles in cyberbullying after controlling the well-established covariates. Six points are worthy of discussion.

First, there were a significant negative associations between age and both traditional and cyber victimization. This indicates that younger students are more vulnerable to victimization. This finding is in agreement with the previous studies (Ševčíková and Šmahel, 2009; Zalaquett and Chatters, 2014) which showed that younger students were more often the victims in comparison to older students. To explain this, Smith et al. (1999) suggest that older students acquire social skills and coping techniques that help them to deal with bullying victimization more practically and successfully.

Second, findings of the present study demonstrated that females were higher on both traditional and cyber victimization than males, while males were higher on the perpetration of both traditional and cyberbullying. These findings are in agreement with the existing literature on traditional bullying (Whitney and Smith, 1993; Yang et al., 2006), that reported greater involvement of males in traditional bullying than females. Similarly, with respect to cyberbullying and victimization, our results corroborate the findings of previous studies on university students that reported greater cyber victimization among females (Faucher et al., 2014; Webber and Ovedovitz, 2018). Further, findings are also consistent with earlier studies carried out in Asian countries that demonstrated that male university students were more likely involved in cyberbullying perpetration than females (Dilmac, 2009; Akbulut and Eristi, 2011).

Third, it was found that females reported three times higher cyber victimization than traditional victimization. Additionally, males reported two times more perpetration of cyberbullying than the perpetration of traditional bullying. This finding is in contrast to existing research on school students (Kowalski and Limber, 2013; Olweus and Limber, 2018), which reported greater involvement of students in traditional bullying. This inconsistency may be explained in terms of differences between school and university students. Generally, university students have 24/7 access to ICT while school children usually have limited and monitored access to technology by parents or school staff. Therefore higher involvement of university students in cyberbullying might be due to greater access, less parental oversight, and more frequent use of technology.

Further, less involvement of university students in traditional bullying in comparison to cyberbullying may be due to the perception of greater accountability, and consideration of the norms of face-to-face interactions in the traditional context in comparison to cyber context in which norms are unclear. Another possible explanation might be the liberating environment (Erdur-Baker, 2010) and perception of the greater anonymity of the digital world (Betts, 2016) that can enhance the involvement of students in cyberbullying.

Fourth, findings confirm the association of spending more time on the Internet and particularly on SNS was associated with an increase in cyberbullying (Zhou et al., 2013; Navarro et al., 2017) and cyber victimization (Sengupta and Chaudhuri, 2011). Researchers such as Betts (2016) explains this association in terms of mere exposure effect; more exposure to technology and online spaces increase the probability of involvement in cyberbullying and victimization.

Fifth, findings demonstrated a significant negative association of GSE with the perpetration of cyberbullying which is consistent with the previous studies (Wong et al., 2014; Eden et al., 2016) which support this finding and indicates that GSE serves as a protective factor to reduce the involvement in cyberbullying perpetration.

Sixth, we tested the comparative role of both general and ICT self-efficacy in predicting the involvement of students as cyber-bullies, cyber-victims, and cyber bully victims in comparison to un-involved students after controlling the effect of covariates. Results showed that ICT self-efficacy significantly decreased the probability of being a cyber-victim and increased the probability of being a cyber-bully. No significant role of GSE was found in predicting the involvement of participants in any role of cyberbullying.

Finally, these findings suggest that ICT self-efficacy is a more important factor with respect to involvement in cyberbullying. Regarding cyber victimization, findings are in agreement with Mishna et al. (2012), who suggested that cyber victims were not aware of the skills related to online safety. Findings for the role of ICT in the involvement of cyberbullying perpetration seems to be consistent with other research which found the positive association between ICT self-efficacy and perpetration of cyberbullying (Xiao and Wong, 2013; Musharraf et al., 2018), and those who reported significant association of cyberbullying perpetration with computer skills (Walrave and Heirman, 2011), and online expertise (Livingstone et al., 2011). A possible explanation for these results may be that those who choose to cyberbully others need skills and ICT self-efficacy may failitate enacting their aggression. For example, perpetrators can conceal their identities, and remove digital footprints of their negative online behaviors by using such skills. Additionally, they may feel safe if they realize their target does not have such skills to identify them or retaliate.

### Implications

Overall, these findings have important implications for developing and enhancing interventions with respect to the inclusion of ICT related skills. Prevention programs might incorporate hands-on practice as well as demonstrations to enhance ICT self-efficacy with a special focus on teaching online safety and security-related skills. Additionally, teaching of such skills should also be incorporated in awareness-raising campaigns and anti-cyberbullying intervention programs. It seems that those inclined to cyberbullying others acquire those skills, but those who may be targeted would benefit from specific instruction on those skills. Moreover, ICT self-efficacy may contribute to the power imbalance between cyberbullying and cyber-victim; ensuring all students have both skills and confidence in their ability to use those skills might diminish the power differential and reduce cyberbullying. It is also important to note that ICT self-efficacy has emerged as a valid predictor of involvement in cyberbullying perpetration. Therefore, ICT self-efficacy based interventions require extra care to teach students the effective use of these skills only for protection, and not for creating abuse.

In addition, the higher level of cyberbullying among university students in comparison to traditional bullying indicates the significance of anti-cyberbullying prevention and intervention efforts to combat cyberbullying in higher education institutions. The findings concerning gender differences contribute to the small existing literature with reference to cyberbullying among university students and indicate the influence of gender in the manifestation of bullying and cyberbullying, particularly in developing or South East Asian countries such as Pakistan.

### Limitations and Recommendations

The study contributed to our understanding of the role of ICT self-efficacy in various participant roles in cyberbullying among university students; however, it has certain limitations. The cross-sectional nature of the study limits the external validity of the findings. It is recommended that future research use a longitudinal design to give a better estimation of the predictive role of ICT self-efficacy in cyberbullying and victimization. For instance, tracking changes over a course of longitudinal study may provide more reliable evidence about the role of ICT self-efficacy in cyberbullying. Additionally, examining alternate models involving potential mediators and moderators for the relationship between ICT self-efficacy and cyberbullying victimization would extend our understanding of cyberbullying phenomenon. It is also recommended that future research would investigate how different dimensions of ICT self-efficacy are related with the involvement in different roles in cyberbullying/victimization.

A potential limitation of the study is the reliance on self-report measures. Self-report measurement may prone to under or over reports of bullying/victimization. Though, we controlled the analysis for social desirability in responding, future research may consider multi-respondent design such as peer ratings to counter the potential bias.

With reference to gender differences in the prevalence of traditional and cyberbullying/victimization, it is important to note that females are significantly younger than males and younger age is correlated with cyber victimization. Therefore, these findings may confound with age variable. Though we controlled the confounding effect of age to estimate the unique role of gender in traditional and cyberbullying/victimization, it is further recommended to use comparative groups of male and female to increase the strength of the research design and precision in estimates. Further, there is discrepancy in the proportion of sample concerning gender (39.05% males versus 60.95% females) and this discrepancy may affect the findings. Therefore, future research may include sample with equal proportion of both males and females.

## Data Availability

The datasets generated for this study are available on request to the corresponding author.

## Ethics Statement

This study was carried out in accordance with the recommendations of “American Psychological Association Ethical Code of Conduct by Institutional Review Board of National Institute of Psychology” with written informed consent from all subjects. All subjects gave written informed consent in accordance with the Declaration of Helsinki. The protocol was approved by the IRB-NIP.

## Author Contributions

SM contributed to conceptualization, data curation, visualization, methodology, formal analysis, project administration, and wrote the original draft. SB performed the formal analysis, supervision, validation, writing, review, before and editing. MA-ul-H contributed to supervision and validation. JM performed the formal analysis, writing, review, before and editing.

## Conflict of Interest Statement

The authors declare that the research was conducted in the absence of any commercial or financial relationships that could be construed as a potential conflict of interest.
